# A comparative study of femoral artery and combined femoral and axillary artery cannulation in veno-arterial extracorporeal membrane oxygenation patients

**DOI:** 10.3389/fcvm.2024.1388577

**Published:** 2024-09-18

**Authors:** Na Jin, Xin Pang, Shiyang Song, Jin Zheng, Zhimeng Liu, Tianxiang Gu, Yang Yu

**Affiliations:** Department of Cardiac Surgery, The First Hospital of China Medical University, Shenyang, China

**Keywords:** cardiac surgery, VA-ECMO, cannulation strategy, clinical outcomes, individualized treatment

## Abstract

**Objective:**

Veno-arterial extracorporeal membrane oxygenation (VA-ECMO) is a critical support technique for cardiac surgery patients. This study compares the outcomes of femoral artery cannulation vs. combined femoral and axillary artery cannulation in post-cardiotomy VA-ECMO patients. This study aimed to compare the clinical outcomes of critically ill patients post-cardiac surgery under VA-ECMO support using different cannulation strategies. Specifically, the focus was on the impact of femoral artery (FA) cannulation vs. combined femoral artery and axillary artery (FA+AA) cannulation on patient outcomes.

**Methods:**

Through a retrospective analysis, we compared 51 adult patients who underwent cardiac surgery and received VA-ECMO support based on the cannulation strategy employed—FA cannulation in 27 cases vs. FA+AA cannulation in 24 cases.

**Results:**

The FA+AA group showed significant advantages over the FA group in terms of the incidence of chronic renal failure (CRF) (37.50% vs. 14.81%, *p* = 0.045), preoperative blood filtration requirement (37.50% vs. 11.11%, *p* = 0.016), decreased platelet count (82.67 ± 44.95 vs. 147.33 ± 108.79, *p* = 0.014), and elevated creatinine (Cr) levels (151.80 ± 60.73 vs. 110.26 ± 57.99, *p* = 0.041), although the two groups had similar 30-day mortality rates (FA group 40.74%, FA+AA group 33.33%). These findings underscore that a combined approach may offer more effective hemodynamic support and better clinical outcomes when selecting an ECMO cannulation strategy.

**Conclusion:**

Despite the FA+AA group patients presenting with more preoperative risk factors, this group has exhibited lower rates of complications and faster recovery during ECMO treatment. While there has been no significant difference in 30-day mortality rates between the two cannulation strategies, the FA+AA approach may be more effective in reducing complications and improving limb ischemia. These findings highlight the importance of individualized treatment strategies and meticulous monitoring in managing post-cardiac surgery ECMO patients.

## Introduction

Following cardiac surgery, some patients may experience respiratory failure or low cardiac output due to prolonged cardiopulmonary bypass use, necessitating additional supportive measures (1–4). Veno-arterial extracorporeal membrane oxygenation (VA-ECMO) has become a crucial life support technology, particularly for patients where traditional management strategies fail to maintain adequate cardiac output due to the complexity of the surgery or poor myocardial protection (5–9). After cardiac surgery, especially in complex cases, additional circulatory support is often needed, including VA-ECMO and post-cardiotomy ECMO (PC-ECMO) (10–12).

Research has shown that peripheral cannulation, such as femoral artery cannulation, results in lower mortality rates than central arterial cannulation (13–15). However, femoral cannulation alone may not be sufficient in certain conditions. Adding an axillary catheter can optimize hemodynamic support and balance blood flow to both the upper and additionally, patients may encounter cardiac arrest or severe arrhythmias during or after surgery, where ECMO temporarily replaces cardiac and pulmonary function to maintain circulation and oxygenation until cardiac function is restored (16–19). ECMO also provides crucial support in low cardiac output syndrome, where the heart's pumping efficiency is inadequate (20–22).

Although some studies suggest that ECMO has not significantly improved survival rates in adult respiratory failure (23), its application in post-cardiac surgery patients has shown promising therapeutic effects, attributed to advancements in ECMO technology, professional training of medical staff, and refined clinical protocols (24–30). Previous studies have indicated that the timing and strategy of ECMO implantation significantly impact patient prognosis, with postoperative implantation associated with higher complications and mortality rates compared to intraoperative implantation (31–34).

This study compares the efficacy and safety of two cannulation strategies in VA-ECMO support post-cardiac surgery: femoral artery (FA) cannulation and combined femoral and axillary artery (FA+AA) cannulation. The dual cannulation strategy aims to provide more comprehensive hemodynamic support by improving blood perfusion and reducing complications arising from hemodynamic instability (35). Although this method may increase the risk of ECMO-related complications, its potential benefits in improving patient survival rates and enhancing postoperative recovery make it worthwhile.

Given the critical role of VA-ECMO in post-cardiac surgery patients, this study explores how different cannulation strategies affect treatment efficacy and prognosis. Traditional ECMO is primarily conducted through femoral artery cannulation, but this approach sometimes encounters limitations in achieving balanced blood perfusion, especially in patients requiring optimized blood flow to both the upper body and brain. Therefore, we introduced an innovative cannulation strategy – combined femoral and axillary artery cannulation (FA+AA) – to enhance blood distribution balance and therapeutic efficacy. Dual cannulating the femoral and axillary arteries allows for a more even blood flow distribution, effectively reducing complications such as North-South syndrome and improving overall patient outcomes.

This study compares the outcomes of different percutaneous arterial cannulation strategies post-cardiac surgery. It provides critical insights into how cannulation strategy impacts patient prognosis, recovery, complication risks, and long-term outcomes, guiding clinicians in selecting more precise and individualized treatment strategies for optimal ECMO management.

## Materials and methods

### Sample and experimental setup

This study is a retrospective cohort analysis aiming to examine the population of adult patients who received VA-ECMO support following cardiac surgery between 2019 and 2022. A total of 59 patients underwent cardiac surgery during the study period. During data cleaning, 8 patients were excluded due to missing data and the application of specific cannulation strategies. These exclusions included 2 pediatric patients with congenital heart disease and 6 adult patients who had undergone a new center cannulation strategy. Ultimately, data from 51 adult patients were included in the statistical analysis.

**Figure 1:**
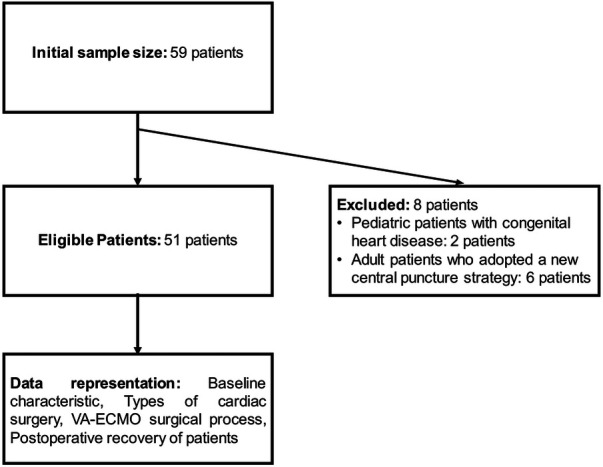
Flow chart for inclusion and exclusion of samples from patients undergoing VA-ECMO after cardiac surgery.

The sample size was determined based on 20 times the number of research variables, ensuring sufficient data for reliable statistical analysis. We recognized the necessity of strict selection and exclusion criteria to ensure data quality and a thorough data completeness assessment. To meticulously document and evaluate each patient's clinical trajectory and the efficacy of VA-ECMO treatment, we collected data on patients’ baseline characteristics (such as age, gender, underlying health conditions, and cardiac history), types and complexity of cardiac surgeries, duration and complications related to VA-ECMO support, and postoperative recovery, including survival rates and hospital stay durations. Reasons for exclusion and basic patient information were also documented for future analysis (Figure 1).

Furthermore, all data were extracted from the electronic medical records system of The First Hospital of China Medical University and independently verified by two researchers to minimize data entry errors. All statistical analyses were performed using the latest version of statistical software packages, encompassing descriptive statistics, survival analysis, and risk comparisons. Before analysis, the First Hospital of China Medical University Ethics Committee approved all research activities, and patient information was handled in strict compliance with data protection regulations. Potential biases and confounding factors were addressed through multivariate analysis adjustments.

### ECMO principles and cannulation techniques

**Figure 2:**
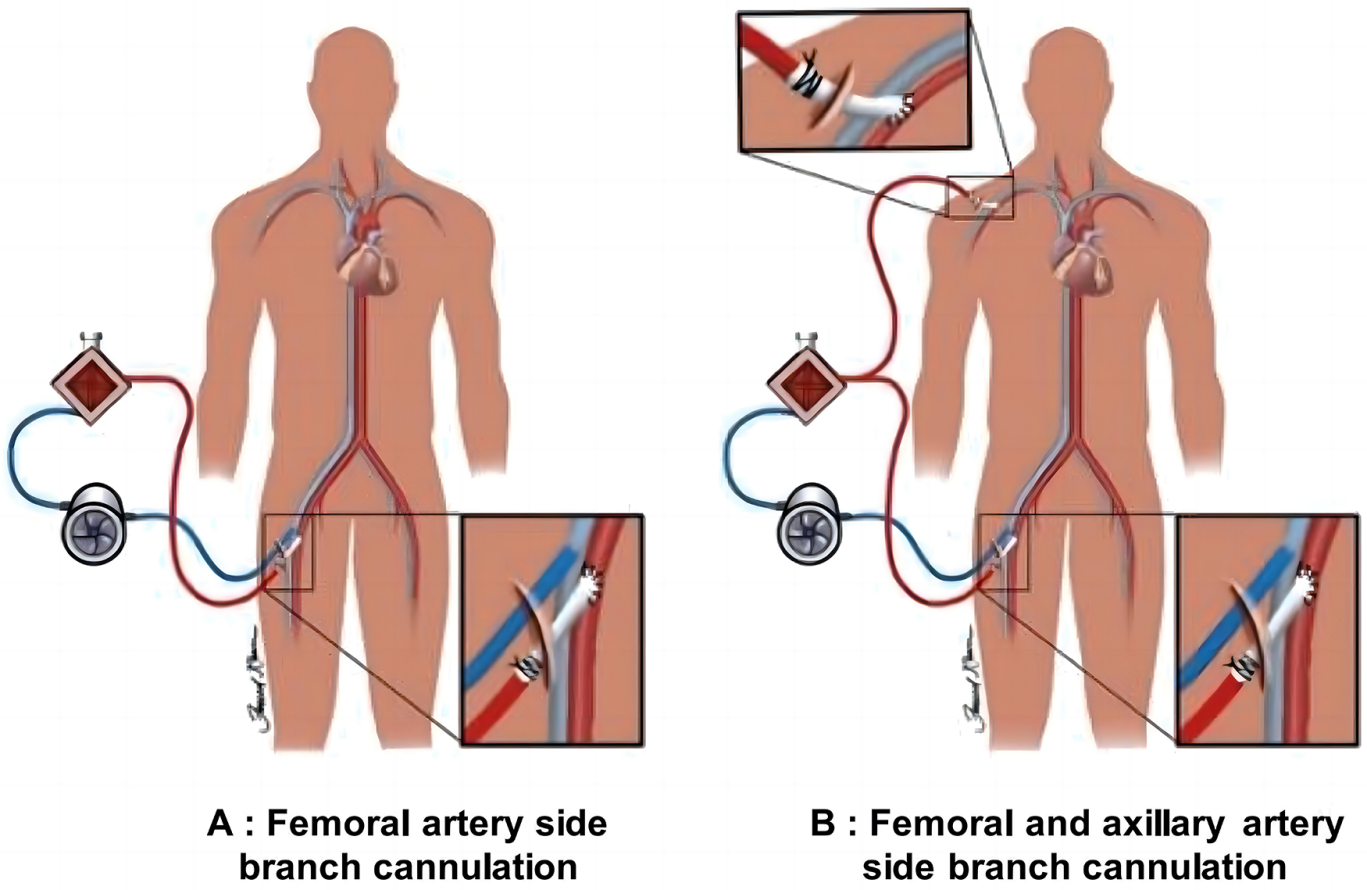
Schematic diagram of ECMO cannulation strategies. **(A)** Illustration of connecting a Dacron vascular graft to the FA via an inguinal incision for arterial perfusion. The graft provides an essential arterial access point for ECMO to support circulation in post-cardiac surgery patients. **(B)** Shows the process of connecting a Dacron graft to the FA and AA using an end-to-side anastomosis technique, enhancing blood perfusion in patients with perioperative low cardiac output syndrome.

In this study, all patients, including intraoperative and postoperative cases, were supported by ECMO to assist circulation. This intervention was necessary as some patients exhibited low cardiac output syndrome during the perioperative period, which could not be maintained solely by high-dose vasopressors or conventional intra-aortic balloon pump (IABP) support. The dual arterial cannulation strategy, including both femoral and axillary artery cannulation, was performed simultaneously with ECMO insertion to ensure immediate and effective support. The FA and vein were exposed through an inguinal incision in the FA cannulation group. An 8 mm Dacron vascular graft was then connected to the FA using an end-to-side anastomosis technique for arterial perfusion (Figure 2A). Venous drainage was achieved through direct cannulation of the femoral vein. For the FA+AA group, both the FA and AA were exposed, and a similar end-to-side anastomosis technique was used to attach an 8 mm Dacron graft to these arteries for arterial perfusion (Figure 2B).

Furthermore, we observed five patients with Type A aortic dissection requiring ECMO support for central arterial cannulation. These patients were included in the analysis group utilizing a unique central cannulation technique, distinct from the main FA and FA+AA groups. In these patients, the fourth branch of the artificial blood vessel was utilized as the arterial access point for ECMO, representing a novel method of ECMO support for patients following complete arch repair with a four-branch artificial blood vessel. To comprehensively analyze these critical aspects, we collected data on the need for left ventricular venting and the occurrence of North-South syndrome during ECMO support.

We adhered strictly to the principles and catheterization techniques during the ECMO implementation. Assessment of indications, selection of modes, evaluation of risks, collaboration of multidisciplinary teams, and close monitoring and management of patients are indispensable steps when implementing ECMO. We have included data and discussion on the need for left ventricular venting and the occurrence of North-South syndrome to provide a comprehensive analysis of these critical aspects. In this study, our focus was on the intracatheter strategy for veno-arterial extracorporeal membrane oxygenation (VA-ECMO), particularly the femoral artery (FA) and combined femoral artery and axillary artery (FA+AA) approaches. These two strategies were selected based on their effectiveness in providing necessary hemodynamic support.

Additionally, we have included data and discussion on the need for left ventricular venting and the occurrence of North-South syndrome to provide a comprehensive analysis of these critical aspects. Catheterizing through the FA, the most common method, is suitable for most post-cardiac surgery patients. On the other hand, FA+AA catheterization utilizes both the FA and AA simultaneously to optimize hemodynamic management, especially in patients where a single FA catheter may not provide adequate blood perfusion. We meticulously documented the implementation process of these strategies, including necessary equipment setup, monitoring, and management measures, to ensure the research's integrity and clinical applicability. To ensure accuracy, the catheter insertion process was conducted under aseptic conditions, typically guided by ultrasound and/or fluoroscopy.

**Figure 3:**
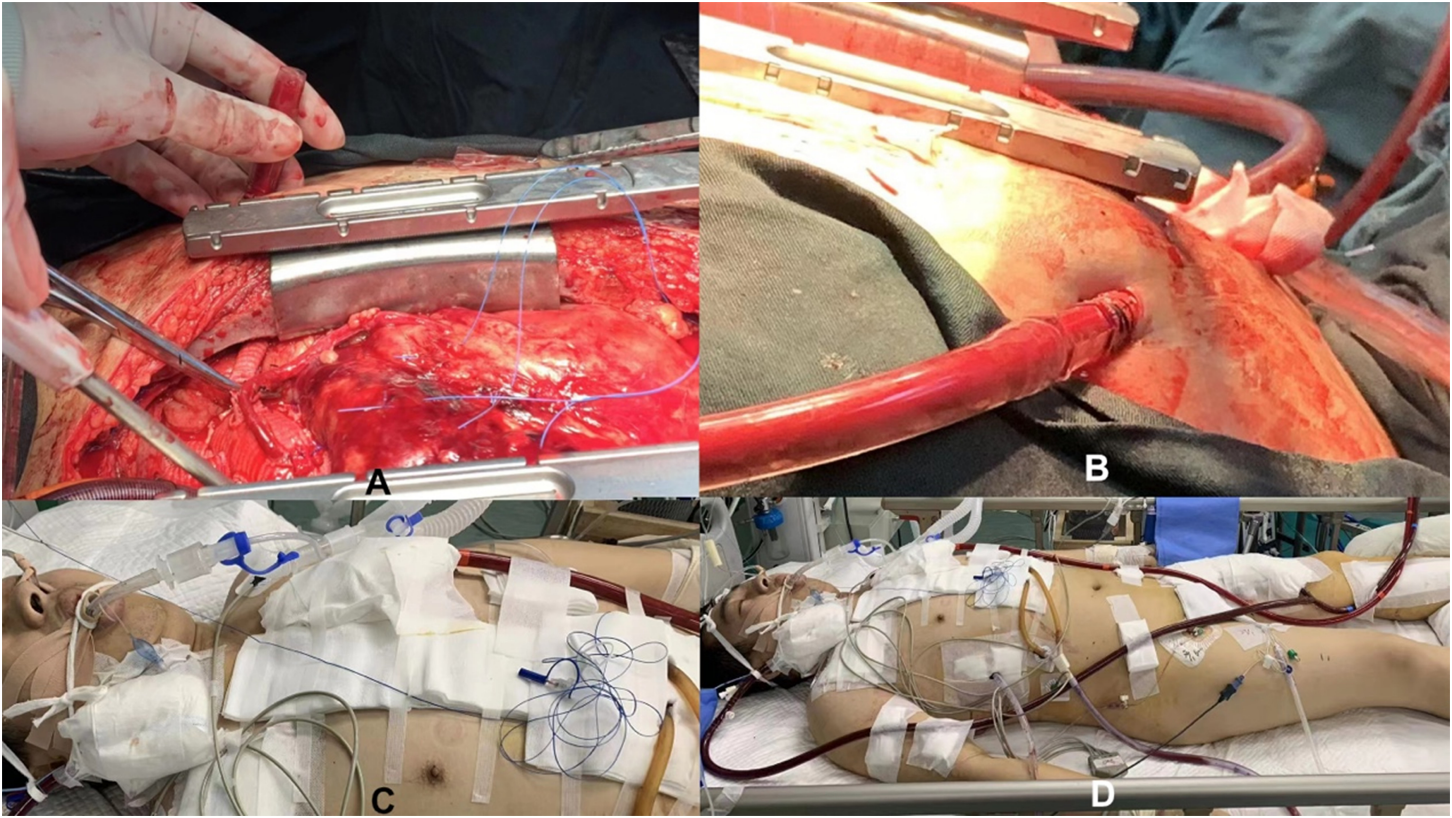
Schematic diagram of central artery cannulation technology supported by ECMO during the perioperative period. **(A,B)** Illustrates central artery cannulation technology during surgery supported by ECMO; **(C,D)** Shows central artery cannulation technology after surgery supported by ECMO.

Additionally, patients under ECMO support usually require anticoagulation therapy to prevent clot formation. Confirming and maintaining catheter positions are crucial to ensure smooth treatment progression and reduce the risk of complications. Ultimately, when the patient's condition permits, we cautiously evaluate and prepare for decannulation, gradually reducing ECMO support until complete removal (Figure 3).

### Statistical variables

All relevant data for this study were sourced from the patient information collection database at our Cardiac Center. The study encompasses variables across five categories:

I. Baseline information: This includes gender, age, diagnosis, height, weight, body surface area (BSA), body mass index (BMI), smoking status, alcohol consumption, hypertension, diabetes, chronic lung disease, peripheral vascular disease (PVD), cerebral infarction (CI), hepatic insufficiency (HI), chronic renal failure (CRF), preoperative atrial fibrillation (preoperative AF), repeat surgeries, emergency surgeries, and preoperative hemofiltration.
II. Intraoperative parameters: These consist of extracorporeal circulation duration, myocardial protection methods, myocardial ischemia duration, occurrence of circulatory arrest, duration of circulatory arrest, mean arterial pressure (MAP) during extracorporeal circulation, ultrafiltration occurrence, ultrafiltration volume, and oxygen consumption (VO2) (36, 37).
III. Laboratory indices on the first postoperative day: These include platelet count, white blood cell count, hemoglobin (Hb), C-reactive protein (CRP), brain natriuretic peptide (BNP)/N-terminal pro-b-type natriuretic peptide (NT-proBNP), neutrophil ratio (NE%), troponin I (TnI), creatine kinase (CK-MB), creatinine (Cr), albumin (ALB), alanine aminotransferase (ALT), lactate (Lac), fluid infusion volume, fluid output volume, urine output, and drainage volume (38). We monitored and recorded lactate levels during and after ECMO support to comprehensively assess organ perfusion.
IV. ECMO period indices: These include platelet count, white blood cell count, CRP, BNP/NT-proBNP, NE%, TnI, CK-MB, Cr, ALB, ALT, Lac, fluid infusion volume, fluid output volume, urine output, drainage volume, and ECMO cannulation strategy.
V. Outcome assessment: This covers the occurrence of postoperative left heart failure, postoperative right heart failure, use of IABP, repeat surgeries, tracheotomy, repeat tracheotomy, dialysis, arrhythmias, days post-surgery with ECMO support, duration of ECMO support, sepsis, limb ischemia, retroperitoneal hematoma, abdominal complications, transient neurological complications, permanent neurological complications, and in-hospital mortality (39).

### Statistical analysis

This study utilized SPSS software (version 26.0.0.2) for statistical analysis. The Kolmogorov-Smirnov test determined the conformity of continuous variables to a normal distribution. Continuous variables following a normal distribution are presented as mean ± standard deviation, while non-normally distributed variables are represented by the median (and interquartile range). Depending on the distribution characteristics of variables, the Student's *t*-test or the Mann-Whitney *U* test was employed for univariate analyses. It should be noted that with larger sample sizes, even when data trends toward a normal distribution, a *P*-value below 0.05 from standard tests may be obtained, in which case the *t*-test is appropriate. The categorical variables were compared through Pearson's chi-square test or Fisher's exact test, depending on the size of the most minor expected count in the contingency table. This study's results with a *P*-value less than 0.05 were considered statistically significant.

## Results

### Comparison of baseline characteristics of patients

This study analyzed 51 adult patients who underwent cardiac surgery and received VA-ECMO support, divided into two groups: the femoral artery (FA) group with 27 cases and the combined femoral and axillary artery (FA+AA) group with 24 cases. Baseline characteristics comparison showed no statistically significant differences between the two groups in terms of gender, age, height, weight, body surface area (BSA), and body mass index (BMI) (all *P* > 0.05). Specifically, the incidence of chronic renal failure (CRF) was 37.50% in the FA+AA group and 14.81% in the FA group (*P* = 0.045). The preoperative blood filtration demand was 37.50% in the FA+AA group and 11.11% in the FA group (*P* = 0.016) (Table 1). The occurrence rate of Stanford type A aortic dissection was 50.00% in the FA+AA group and 37.04% in the FA group (*P* = 0.0148). The two groups had no significant differences in other disease categories, including Stanford type B aortic dissection, coronary artery disease, and valve disease (Table 2).

**Table 1:** Baseline demographic and clinical characteristics of patients.

Variables	FA	FA+AA	*P* value
Male	20 (74.07%)	14 (58.33%)	1
Age (y)	54.67 ± 11.34	56.05 ± 14.40	0.714
Hight (cm)	170.48 ± 8.58	169.67 ± 9.18	0.767
Weight (kg)	75.74 ± 16.42	77.60 ± 13.63	0.718
BSA	1.86 ± 0.26	1.90 ± 0.21	0.562
BMI	25.80 ± 3.68	27.18 ± 2.06	0.205
Smoke	8 (29.63%)	4 (16.67%)	0.517
Drunk	5 (18.52%)	3 (12.50%)	1
Hypertension	9 (33.33%)	10 (41.67%)	0.368
Diabetes	5 (18.52%)	2 (8.33%)	0.682
Chronic lung disease	1 (3.70%)	0 (0.00%)	1
PVD	2 (7.41%)	1 (4.17%)	1
Cerebral infarction (CI)	6 (22.22%)	7 (29.17%)	0.511
Hepatic insufficiency (HI)	3 (11.11%)	7 (29.17%)	0.073
CRF	4 (14.81%)	9 (37.50%)	0.045
Preoperative AF	5 (18.52%)	3 (12.50%)	1
Secondary surgery	6 (22.22%)	3 (12.50%)	0.713
Emergency surgery	20 (74.07%)	17 (70.83%)	0.481
Preoperative hemofiltration	3 (11.11%)	9 (37.50%)	0.016

FA, femoral artery; FA+AA, femoral cannulation+axillary artery; BSA, body surface area; BMI, body mass index; PVD, peripheral vascular disease; CI, cerebral infarction; HI, hepatic insufficiency; CRF, chronic renal failure; Preoperative AF, preoperative atrial fibrillation. *P* < 0.05 indicates that the result is statistically significant; *P* ≥ 0.05 indicates that the results are not statistically significant.

**Table 2:** Patient diagnostic categories.

Variables	FA	FA+AA	*P* value
Dissection type A	10 (37.04%)	12 (50.00%)	0.0148
Dissection type B	1 (3.70%)	0 (0.00%)	0.5
Coronary heart disease	8 (29.63%)	6 (25.00%)	0.546
Valve disease	7 (25.93%)	5 (25.00%)	0.767
Others	1 (3.70%)	1 (4.17%)	1

*p* < 0.05: indicates that the result is statistically significant. *P* ≥ 0.05: indicates that the results are not statistically significant.

### Comparison of intraoperative parameters

This study analyzed post-cardiac surgery patients utilizing different cannulation strategies (FA and FA+AA). The average myocardial ischemia time was 112.00 ± 47.31 min in the FA+AA group and 82.29 ± 44.02 min in the FA group, showing a statistically significant difference between the two groups (*P* = 0.046). The observed longer myocardial ischemia time in the FA+AA group was mainly attributed to the more complex surgical procedures involved, which may require a longer aortic cross-clamp time and is not directly related to the timing of ECMO cannulation. Our study also observed that the myocardial ischemia time was indeed prolonged in the FA+AA group compared to the FA group (112.00 ± 47.31 min vs. 82.29 ± 44.02 min), primarily due to the complexity of the FA+AA procedure. However, we also noted that patients undergoing FA+AA cannulation had better clinical benefits regarding lactic acid levels and limb ischemia improvement than the FA group. It suggests that although this method is time-consuming, it provides better hemodynamic support and improved clinical outcomes. Other surgical parameters such as cardiopulmonary bypass (CPB) time, cross-clamp time, VO2 levels, and maximum mean arterial pressure (MAP) during the first and second CPB runs did not show statistically significant differences between the two groups (all *P* > 0.05). There was also no significant difference in the choice of myocardial protection strategy between the two groups, with a slightly higher proportion of retrograde and antegrade cardioplegia (Method 2) used in the FA+AA group compared to the FA group, albeit without statistical significance (Table 3).

**Table 3:** Surgical procedure parameters and outcomes.

Variables	FA	FA+AA	*P* value
CPB time	187.13 ± 86.66	207.11 ± 111.42	0.517
Circulation arrest exists	12	12	0.353
Circulation arrest time	8.09 ± 8.23	10.56 ± 8.33	0.349
Myocardial ischemia time	82.29 ± 44.02	112.00 ± 47.31	0.046
Myocardial protection method1#	9	5	0.44
Myocardial protection method2#	12	13	
Myocardial protection method3#	2	0	
VO2	70.66 ± 10.55	68.64 ± 6.22	0.492
Max MAP in 1st time CPB	77.65 ± 7.83	81.78 ± 5.85	0.07
Max MAP in 2nd time CPB	55.48 ± 9.70	59.28 ± 8.71	0.201
Average MAP	58.89 ± 22.86	63.48 ± 22.32	0.499
Ultrafiltration exists	22	18	0.498
Ultrafiltration volume	2,900 ± 1,602.08	3,116.67 ± 1,368.23	0.652

CPB, cardiopulmonary bypass; VO2, Oxygen consumption; MAP, mean arterial pressure. Myocardial protection method1#: retrograde perfusion; Myocardial protection method2#: retrograde perfusion + anterograde perfusion; Myocardial protection method3#: no perfusion; *P* < 0.05 indicates that the result is statistically significant; *P* ≥ 0.05 indicates that the results are not statistically significant.

### Laboratory indices before and during ECMO treatment

This study compared patients under two different cannulation strategies, FA and FA+AA, regarding laboratory parameters before and after ECMO treatment. The blood sample collection time before ECMO treatment is before the initiation of ECMO, and the blood sample collection time after ECMO treatment is on the first day following the ECMO procedure. Before ECMO therapy, the mean platelet count in the FA+AA group was 82.67 ± 44.95, compared to 147.33 ± 108.79 in the FA group, while the creatinine levels were 151.80 ± 60.73 and 110.26 ± 57.99 in the FA+AA and FA groups, respectively (with *P* values of 0.014 and 0.041) (Table 4). During ECMO treatment, major laboratory indicators like platelets, white blood cells, hemoglobin, neutrophil percentage, C-reactive protein, B-type natriuretic peptide, N-terminal pro-B-type natriuretic peptide, creatinine, albumin, aspartate aminotransferase, fluid input, fluid output, urine output, drainage volume, troponin I and creatine kinase-MB showed *P* values higher than 0.05 between the two groups (Table 5). We have acknowledged the importance of lactate levels as an indicator of organ perfusion and have included follow-up data on lactate levels to provide a comprehensive assessment of organ perfusion during and after ECMO support. Although there was no statistical difference between the FA and FA+AA groups, it can be observed that the FA group had an increasing trend in lactate levels during treatment compared to before treatment, while the FA+AA group showed no significant change in lactate levels, indicating that organ perfusion in the FA+AA group may have been relatively improved compared to the FA group.

**Table 4:** Laboratory parameters before ECMO therapy.

Variables	FA	FA+AA	*P* value
Platelet	147.33 ± 108.79	82.67 ± 44.95	0.014
Leukocyte	14.56 ± 6.95	15.64 ± 4.82	0.6
HB	110.79 ± 28	102.67 ± 29.96	0.396
NE%	72.31 ± 22.89	82.12 ± 9.71	0.073
CRP	159.63 ± 108.89	107.93 ± 60.57	0.238
BNP	511.33 ± 529.01	741.75 ± 502.58	0.478
NT-proBNP	2,070.91 ± 1,495.39	7,117.32 ± 11,832.84	0.239
Cr	110.26 ± 57.99	151.80 ± 60.73	0.041
ALB	36.18 ± 5.34	37.57 ± 4.10	0.394
ALT	380 ± 873.30	358.40 ± 350.73	0.928
Infusion volume	1,796 (IQR: 1,200–2,200)	1,060 (IQR: 800–1,500)	0.13
Liquid output	1,148.63 (IQR: 800–1,600	1,966.37 (IQR: 1,300–2,500)	0.046
Urine output	361.85 (IQR: 300–500)	1,059.74 (IQR: 800–1,200)	0.048
Drainage	168.15 (IQR: 100–200)	158.42 (IQR: 120–180)	0.897
Tnl	17.84 ± 26.87	22.94 ± 28.53	0.588
CKmb	55.04 ± 94.88	56.49 ± 83.33	0.964
Lactate (Lac)	10.9 ± 5.38	12.36 ± 4.68	0.448

HB, hemoglobin; NE%, neutrophils radio; CRP, C-Reactive protein; BNP, brain natriuretic peptide; NT-proBNP, N-terminal pro-b-type natriuretic peptide; Cr, Creatinine; ALB, Albumin; ALT, Alanine Aminotransferase; Tnl, Troponin I; CKmb, Creatine Kinase-MB; Lac, Lactate. *P* < 0.05 indicates that the result is statistically significant; *P* ≥ 0.05 indicates that the results are not statistically significant.

**Table 5:** Laboratory parameters during ECMO therapy.

Variables	FA	FA+AA	*P* value
Platelet	85.73 ± 59.69	66.78 ± 47.61	0.282
Leukocyte	14.03 ± 6.09	11.50 ± 5.38	0.176
HB	87.95 ± 18.03	79.34 ± 22.12	0.182
NE%	78.25 ± 22.86	83.23 ± 17.31	0.45
CRP	115.46 ± 102.24	136.50 ± 126.04	0.68
BNP	654.56 ± 491.14	962.6 ± 793.81	0.382
NT-proBNP	4,138.55 ± 3,018.65	3,562.27 ± 2,391.71	0.657
Cr	145.55 ± 65.96	136.23 ± 51.61	0.634
ALB	30.98 ± 6.69	34.73 ± 6.38	0.08
ALT	446.83 ± 787.04	734.94 ± 955.07	0.302
Infusion volume	4,518.97 (IQR: 3,000–5,000)	4,894.66 (IQR: 3,500–5,500)	0.608
Liquid output	3,768.15 (IQR: 3,000–4,500)	3,516.00 (IQR: 2,500–4,000)	0.721
Urine output	1,909.93 (IQR: 1,000–2,000)	1,711.75 (IQR: 800–1,500)	0.771
Drainage	386.48 (IQR: 200–400)	305.75 (IQR: 100–300)	0.506
Tnl	27.73 ± 28.42	27.22 ± 22.36	0.952
CKmb	88.69 ± 108.16	95.65 ± 102.18	0.841
Lactate (Lac)	12.36 ± 5.15	12.95 ± 4.47	0.689

HB, Hemoglobin; NE%, neutrophil percentage; CRP, C-Reactive protein; BNP, brain natriuretic peptide; Cr, Creatinine; ALB, Albumin; ALT, Alanine Aminotransferase; Urine output, drainage 24 h; Tnl, Troponin T; CKmb, Creatine Kinase-MB; Lac, Lactate. All measurement data was performed at the first day after ECMO treatment; *P* < 0.05 indicates that the result is statistically significant. *P* ≥ 0.05 indicates that the results are not statistically significant.

We compared lactate levels in patients under different cannulation strategies (FA and FA+AA) during and after ECMO treatment. Before treatment, the average lactate level in the FA group was 2.1 ± 0.6 mmol/L, while in the FA+AA group, it was 1.9 ± 0.5 mmol/L (*P* > 0.05). During ECMO treatment, lactate levels in the FA group significantly increased to 3.4 ± 1.0 mmol/L, whereas the FA+AA group maintained stable levels at 2.0 ± 0.7 mmol/L (*P* < 0.05). This result indicates that organ perfusion may be relatively improved in the FA+AA group compared to the FA group.

### Post-ECMO support comparison

In this study, we focused on the clinical outcomes of patients after weaning off extracorporeal membrane oxygenation (ECMO) support, with particular attention to comparing different cannulation strategies (FA and FA+AA) (Table 6). Regarding the improvement in limb ischemia, the FA+AA group demonstrated significantly greater progress than the FA group (*P* = 0.029). Additionally, the inpatient mortality rates were 40.74% for the FA group and 40% for the FA+AA group, indicating no significant difference in the 30-day postoperative mortality rates between the two strategies. Other key clinical indicators such as postoperative left ventricular failure, right ventricular failure, intra-aortic balloon pump (IABP) use, reoperation, tracheostomy tube use, dialysis, arrhythmias, post-ECMO days of use, duration of ECMO support, sepsis, retroperitoneal hematoma, abdominal complications, as well as temporary and permanent neurological sequelae, showed *P* values above 0.05 between the two groups, suggesting no significant differences in these clinical indicators between the two cannulation strategies. During the ECMO support period, 12 patients (23.5%) required left ventricular venting due to elevated left atrial pressure and pulmonary edema. North-South syndrome was observed in 8 patients (15.7%) in the FA group and 2 patients (8.3%) in the FA+AA group. These findings highlight the differences in hemodynamic management between the two cannulation strategies.

**Table 6:** Postoperative outcomes and complications in ECMO patient groups.

Variables	FA	FA+AA	*P* value
Postoperative left heart failure	14 (51.85%)	10 (41.67%)	1
Postoperative right heart failure	22 (81.48%)	17 (70.83%)	1
IABP use	7 (25.93%)	6 (25.00)	1
Reoperation	8 (29.63%)	4 (16.67%)	0.517
Tracheostomy Tube	1 (3.70%)	0 (0.00%)	1
Tracheostomy Tube Reoperation	3 (11.11%)	0 (0.00%)	0.251
Dialysis	11 (40.74%)	13 (54.17%)	0.142
Arrhythmias	23 (85.19%)	16 (66.67%)	0.707
Postoperative days when using ECMO	1.15 ± 2.13	1.7 ± 2.56	0.424
ECMO duration	5.19 ± 5.39	8.45 ± 7.63	0.092
Septicemia	1 (3.70%)	0 (0.00%)	1
Upper limb ischemia	13 (48.15%)	3 (12.50%)	0.029
Lower limb ischemia	10 (37.04%)	2 (8.33%)	0.048
Retroperitoneal hematoma	0 (0.00%)	0 (0.00%)	1
Abdominal complications	11 (40.74%)	4 (16.67%)	0.206
Ischemic stroke	5 (18.52%)	2 (8.33%)	0.275
Intracranial bleeding	6 (22.22%)	3 (12.50%)	0.350
Seizures	4 (14.81%)	1 (4.17%)	0.174
In-hospital mortality	11 (40.74%)	8 (33.33%)	1

IABP, intra-aortic balloon pump. *P* < 0.05 indicates that the result is statistically significant; *P* ≥ 0.05 indicates that the results are not statistically significant.

## Discussion

Post-cardiac surgery, critically ill patients often experience inadequate tissue perfusion (cardiogenic shock) due to low cardiac output and challenges in maintaining perfusion pressure (40–42). The causes of cardiogenic shock can be diverse, including myocardial injury caused by cardiac arrest, cardiopulmonary bypass, and anesthesia, among other factors (43, 44). These patients typically require high doses of inotropic agents to maintain blood pressure (45–47). Additionally, mechanical assist devices, such as IABP, Impella, ventricular assist devices (VAD), or ECMO, are often necessary. ECMO, in particular, is an effective circulatory support mode widely accepted by doctors and patients in China (48). Depending on the cannulation strategy and application guidelines, ECMO can be categorized into various modes, such as VA, VV, and VAV. The VV mode is more suitable for respiratory failure caused by various reasons. This paper focuses on patients with reduced cardiac output post-cardiac surgery, thus employing the VA mode for ECMO support. This study is a retrospective analysis aimed at observing the differences in short-term improvements in circulatory and physiological conditions, ECMO-related complications, and short-term or long-term clinical outcomes among adult patients using different arterial cannulation strategies.

In VA-ECMO, arterial cannulation methods primarily include direct unilateral FA cannulation, FA branch anastomosis cannulation, a combination of FA and AA cannulation, and FA plus AA branch anastomosis cannulation (49, 50). FA combined with femoral vein cannulation is the most straightforward and feasible method (51–53). However, limb ischemia on the cannulated side is common in elderly patients with pre-existing peripheral vascular disease (54, 55). Initially, we used reverse perfusion catheters (diameter 5–6 Fr) at the distal end of the FA, but this did not significantly alleviate ischemic symptoms in some patients. Thus, we adopted an 8 mm FA prosthetic side anastomosis method, addressing central FA limb ischemia. The placement of femoral arterial (FA) catheters is associated with North-South Syndrome, primarily due to the uneven distribution of blood flow away from the heart towards the lower body from the catheter insertion point. Conversely, radial arterial (AA) catheter insertion may lead to increased afterload, attributable to the catheter's closer proximity to the heart, affecting the hemodynamics of blood return to the heart. It is crucial to differentiate between these two scenarios as they involve distinct physiological mechanisms and potential clinical management strategies (56–59).

Therefore, we further adopted a combined method of femoral artery and axillary artery cannulation (side graft anastomosis). This approach, involving simultaneous cannulation of the femoral artery and axillary artery, allows for a more even distribution of blood flow to the upper and lower parts of the body, effectively reducing the common north-south syndrome and associated complications seen with traditional methods. Additionally, the FA+AA strategy has shown potential advantages in providing extra hemodynamic stability, making it particularly suitable for patients with severely impaired cardiac function. We used a unique method for certain special patients with Type A aortic dissection: externalizing through the fourth 2–3 intercostal space and directly using an artificial vessel as the arterial route connected to ECMO. According to related indicators, this method does not increase surgical time (from skin incision to ECMO operation) and has advantages such as rapidly improving systemic condition, preventing limb ischemia, reducing cerebral complications, and improving short-term or long-term prognosis. This dual cannulation method potentially reduces complications caused by hemodynamic instability and improves physiological oxygenation levels by enhancing blood perfusion. Although this method may increase the risk of ECMO-related complications such as bleeding and vascular injury, its potential benefits in improving patient survival rates and postoperative recovery make it a strategy worth considering.

This study comprehensively analyzed the differences and potential advantages between isolated femoral arterial (FA) catheterization and combined FA+AA catheterization in ECMO support following cardiac surgery. Combined FA+AA catheterization demonstrates significant advantages over isolated FA catheterization in several key aspects. Firstly, the combined use of femoral and axillary arterial catheters provides a more balanced blood distribution, particularly between the upper and lower body, helping to reduce complications resulting from hemodynamic imbalances such as North-South Syndrome. Additionally, FA+AA catheterization enhances perfusion stability through two distinct vascular pathways, offering extra safety measures for patients with severe cardiac dysfunction, especially when facing unpredictable hemodynamic changes. Lastly, this approach, with broader vascular coverage, potentially reduces the risk of local hypoperfusion and limb ischemia, thus decreasing the occurrence of severe vascular-related complications and better managing North-South Syndrome. Considering all factors, combined FA+AA catheterization optimizes hemodynamic management and enhances overall patient care, particularly suitable for patients with significant anatomical challenges or severe cardiac impairment.

For patients with Type A aortic dissection, we typically employ a standardized and uniform approach for ascending aorta replacement, total aortic arch replacement with a four-branch graft, and descending aorta stent implantation. This treatment is critical for these patients due to the acute reconstruction of the distal aorta following the elimination of the primary tear. In patients with multiple tears and involvement of visceral arteries, perfusion to distal organs often remains uncertain. Antegrade perfusion is crucial to ensure no injury to the thoracic segment. Additionally, the blood supply to abdominal organs might benefit more, offering an advantage in aortic reconstruction (60).

Our study group comprised 51 patients who received ECMO implantation at the bedside. Based on ECMO cannulation strategies, data were divided into the FA group (27 patients, 52.94%) and the FA+AA group (24 patients, 47.06%). The results of these two strategies were assessed by comparing multiple parameters across various dimensions.

In our ICU, surgeons manage the ECMO at the bedside, with perfusionists monitoring the pump. Our department's registered nurses are pivotal in assisting physicians and observing and caring for patients throughout the nursing process.

Based on our experience, the establishment, management, and nursing of ECMO primarily encompass four phases: 1. bedside surgery, 2. initiation of the ECMO pump, 3. recovery period under ECMO support, and 4. gradual weaning from ECMO and decannulation.

In the first phase, bedside surgery, ICU nurses partly assume the responsibilities of scrub nurses. Familiarity with surgical instruments and checklists is crucial. Nurses should be adept with aseptic techniques and surgical procedures. Bedside surgery packs need to be readily accessible and prepared for use. When arranging V and A-line catheters, care must be taken to prevent stress injuries. Initial limb diameters should be documented for further assessment. CRRT access is typically pre-set on the ECMO cannulation, requiring vigilance to prevent air embolism.

The second phase involves ECMO initiation. From the start of ECMO, patients begin adapting to ECMO support. Patients’ hemodynamic status undergoes adjustments in the initial hours of ECMO pump activation. Monitoring blood pressure, heart rate or rhythm, and body temperature is essential alongside fluid therapy. Extra attention is required for surgical sites/incisions. Bleeding at anastomotic sites (with hidden hemorrhage) may occur when the pump increases local pressure. Studies have indicated that prone positioning during ECMO nursing can enhance patients’ cardiopulmonary function (61).

During the third phase, the recovery period under ECMO support, patients gradually recuperate once their hemodynamic status stabilizes, each presenting unique issues based on their condition. Typical observations include proper atrial and cardiac output measurements affected by ECMO, blood oxygen saturation on the ear or right hand, and body temperature. Neurological observations are critical due to bleeding risks, hypoxemia, and low perfusion. Peripheral observations around limbs include color, warmth, and pulse. Concurrently, reperfusion lines monitor fluid balance, continuous venovenous hemofiltration (CVVH) connected to the ECMO circuit, urine output and color, and renal function parameters.

The fourth phase involves ECMO weaning and cannulation removal. Nurses should pay extra attention to patients’ conditions post-decannulation. Trends or changes in parameters often prove more significant than numerical values. Post-ECMO, patients’ positioning is vital for redistributing blood volume and lung inflation. Reverse Trendelenburg positioning is more beneficial for patients. Skin conditions should also be reassessed, and further protective plans should be formulated.

The six patients excluded from the statistical analysis of this study were as follows: an adult male for postoperative respiratory failure (pulmonary consolidation due to infection) using VV-ECMO; several pediatric patients underwent surgery with delayed chest closure due to the inability to perform peripheral arterial and venous cannulation; and four patients who underwent aortic dissection surgery and total aortic arch replacement using a four-branched vessel for operation. This branch was passed through the left chest wall via the second or third intercostal space and connected to the arterial end of ECMO, with a femoral venous catheter used on the venous end. This method established and operated ECMO effectively and safely, requiring only ligation and suturing of the arterial end and embedding it into the chest wall.

Our findings showed that during ECMO treatment, lactate levels in the FA+AA group were significantly lower than those in the FA group, indicating that the FA+AA cannulation strategy may be more advantageous in improving organ perfusion. Our study showed that lactate levels were significantly lower in the FA+AA group compared to the FA group, supporting the idea that hyperlactatemia is associated with increased in-hospital mortality in postcardiotomy VA-ECMO patients (62). This finding further supports the potential benefits of the FA+AA strategy in managing patients with low cardiac output syndrome. Our study also observed that the need for left ventricular venting was higher in the FA group than the FA+AA group, which may be attributed to the more stable hemodynamic support the dual cannulation strategy provided. Additionally, the occurrence of North-South syndrome was lower in the FA+AA group, further supporting the potential benefits of this approach in managing complex hemodynamic conditions during ECMO support.

**Figure 4:**
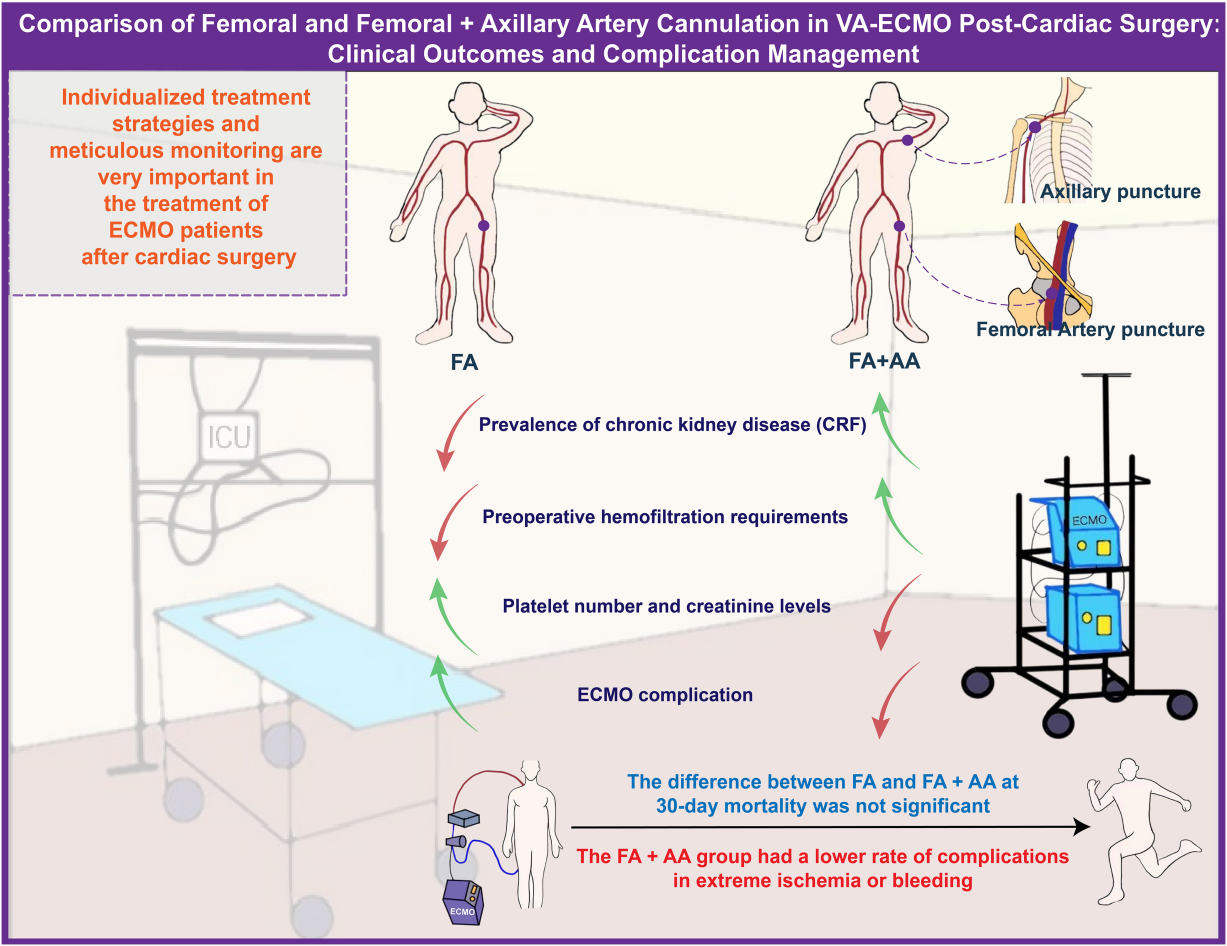
Comparison of FA and FA+AA cannulation in VA-ECMO post-cardiac surgery: clinical outcomes and complication management.

This study compared different arterial cannulation strategies in critically ill patients receiving ECMO support post-cardiac surgery, a topic of significant clinical importance. It was found that patient baseline characteristics, surgical management parameters, laboratory indices before and during ECMO, and performance post-ECMO discontinuation varied depending on the cannulation strategy. Notably, the FA+AA group improved limb ischemia, which is crucial for patient recovery and survival. Scientifically, this research provides valuable information for ECMO treatment in critically ill post-cardiac surgery patients, aiding in understanding which strategy might positively impact patients’ physiological state and recovery. It aids physicians in better selecting the appropriate ECMO cannulation strategy in clinical practice, thereby enhancing treatment effectiveness. Clinically, this study offers guidance for medical teams to manage patients needing ECMO support post-cardiac surgery. It emphasizes the importance of individualized treatment strategies, as patients may respond differently to various cannulation strategies. Furthermore, this study underscores the necessity for meticulous monitoring and intervention of patients’ physiological states before and during ECMO treatment to minimize adverse events (Figure 4).

This study has certain limitations. The sample size is limited to 51 cases, all from our hospital. Future research can expand the sample size and include results from multiple centers to further validate the findings and investigate the differences in responses among various patient subgroups. Additionally, this study only explored the VA-ECMO mode of ECMO, and further research is needed on the application of PC-ECMO and the clinical follow-up of patients after ECMO removal. Future studies could consider using more advanced monitoring techniques and treatment methods and comprehensively following up on patients from admission to discharge to further improve the ECMO treatment outcomes for critically ill patients.

## Conclusion

In conclusion, this study provides important references for optimizing ECMO treatment strategies and is expected to improve the clinical outcomes of critically ill patients after cardiac surgery. This study lays the foundation for further exploration of the impact of different puncture strategies.

## Data Availability

The original contributions presented in the study are included in the article/Supplementary Material, further inquiries can be directed to the corresponding authors.
